# Discovery and rescue of porcine bastroviruses associated with polioencephalomyelitis in domestic pigs

**DOI:** 10.1128/jvi.01130-25

**Published:** 2025-08-18

**Authors:** Nicole Wildi, Stefano Bagatella, Xuanxuan Zhang, Mark C. Hawes, Kara L. D. Dawson, Honglei Chen, Som Walker, Gemma Harvey, Brenda van der Heide, David T. Williams, Andrew Hemphill, Corinne Gurtner, Jianning Wang, Torsten Seuberlich

**Affiliations:** 1 Department of Clinical Research and Veterinary Public Health, University of Bern 27210, Bern, Switzerland; 2 Department of Infectious Diseases and Pathobiology, University of Bern 27210, Bern, Switzerland; 3 Graduate School for Cellular and Biomedical Sciences, University of Bern 27210, Bern, Switzerland; 4 Agriculture Victoria 468590 , Bundoora, Victoria, Australia; 5 CSIRO Australian Centre for Disease Preparedness 64808, Geelong, Victoria, Australia; University of Michigan Medical School, Ann Arbor, Michigan, USA

**Keywords:** neurological disease, metatranscriptomics, emerging infectious diseases, bastrovirus, pigs, encephalitis

## Abstract

Bastroviruses (BastV) are non-enveloped single-stranded positive-sense RNA viruses that have been discovered recently in feces samples of different animals and humans. The non-structural proteins of these viruses show similarities to those of hepevirids (*Hepeviridae*), and the structural proteins exhibit similarities to those of astrovirids (*Astroviridae*). BastVs have been found in fecal samples of mammals, amphibians, and invertebrates, but the association of infection and clinical disease manifestations has not been established. Here, we report the identification of porcine bastroviruses (PoBastV) in central nervous system (CNS) tissue samples of domestic pigs that presented fatal neurological disease in two unrelated disease outbreak scenarios in Australia and Switzerland. Viral metatranscriptomics of formalin-fixed paraffin-embedded (FFPE) CNS tissues identified genomic sequences of two genetically closely related PoBastV strains (PoBastV AUS/2015 and CHE/2022). Genomic RNA of both strains was readily detected by *in situ* RNA hybridization in neurons and glial cells of CNS tissues presenting histopathological lesions, thus supporting a plausible causal relationship between neurotropism and disease. We generated a molecular cDNA clone of PoBastV CHE/2022 and rescued infectious virus by reverse genetics in swine kidney cells (SK6) and further virus passage in intestinal porcine enterocytes (IPEC-J2). We used transmission electron microscopy to demonstrate PoBastV CHE/2022 virions in infected IPEC-J2 cells. These findings pave the way toward PoBastV as one of the etiologies of neurological disease outbreaks. Additionally, they allow studies further elucidating the molecular biology and pathogenesis of emerging BastV infections.

**IMPORTANCE:**

Bastroviruses (BastV) have been discovered recently in feces samples of different animals and humans. To date, BastV infections have not been associated with clinical diseases. Here, we report the identification of porcine BastV (PoBastV) in central nervous system tissue samples of domestic pigs that presented fatal neurological disease in two unrelated disease outbreak scenarios in Australia and Switzerland. This finding supports the hypothesis that PoBastV infections may cause clinical disease. We further rescued infectious PoBastV *in vitro* using the genome sequence data of one neuroinvasive PoBastV strain. With these tools, we can now start deciphering the molecular biology of BastV replication and the interaction of the virus with the host, which will lay the ground for future prophylactic and therapeutic strategies.

## INTRODUCTION

Bastroviruses (basal astroviruses [BastVs]) are non-enveloped positive-stranded (ss+) RNA viruses that were first described in 2016 in human stool specimens in the Netherlands (1). BastV genomes are ~6.5 kb in size and are organized into short 5′ and 3′ untranslated regions (UTRs) and at least two open reading frames (ORFs), with ORF1 coding for non-structural proteins and ORF2 coding for the structural proteins. Notably, the ORF1-encoded proteins are phylogenetically related to the non-structural proteins of members of the family *Hepeviridae,* while the ORF2-encoded proteins are similar to those of viruses of the family *Astroviridae* (1). Hence, BastVs have also been referred to as “hepe-astroviruses.” Both viral families include viruses associated with fatal diseases in humans (2, 3) and animals (4–6). Hepatitis E virus (HEV; *Hepeviridae*) causes hepatitis in humans, and domestic pigs and wild boars are among the main reservoirs for HEV genotypes 3 and 4 (7). Astrovirus infections in mammalian hosts are mostly asymptomatic and restricted to the gastrointestinal tract. However, in humans, they are a main cause of juvenile gastroenteritis. Some astroviruses in humans, mink, ruminants, and pigs have neuroinvasive properties and are associated with neurological disease characterized by a non-suppurative polioencephalomyelitis (8).

To date, BastVs have been found in fecal samples of mammals (9, 10), amphibians (11, 12), and invertebrates (13), but the association of infection and clinical disease manifestations has not yet been established. In pigs, BastV has been reported in feces samples collected from both asymptomatic animals and animals with diarrhea (9, 14).

Here, we identified porcine bastroviruses (PoBastVs) in central nervous system (CNS) samples of pigs with neurological diseases in the context of two unrelated disease situations that occurred in Australia (2015) and Switzerland (2022). By high-throughput sequencing (HTS), we obtained full or near full-length BastV genomes and demonstrated viral neuroinvasion and neurotropism by RNA *in situ* hybridization. Using the genome sequence data of one neuroinvasive BastV strain, we established a reverse genetics system and rescued infectious porcine BastV *in vitro*.

## RESULTS

### Case presentations

In Australia, in 2015, a neurological disease situation occurred on a pig farm in western Victoria with fifty 6-week-old piglets. Of them, one piglet from each of two separate litters over a period of 3 months was affected. Clinical signs included lethargy, ataxia, and hind limb paresis, progressing to recumbency over 24 h. One diseased pig was euthanized (pig AUS/1) and pathological examination was performed. Blood and brain samples tested negative using reverse transcription-quantitative PCR (RT-qPCR) for pseudorabies virus, classic swine fever virus, Hendra virus, Nipah virus, sapelovirus, teschovirus, malignant catarrhal fever virus (ovine herpesvirus 2), and orthoflaviviruses. Test of the piglet serum for antibodies against pseudorabies virus using enzyme-linked immunosorbent assay was negative. Virus isolation was attempted with brain and spinal cord tissue using Vero, PK15a, and ST (pig testis) cell lines. No virus was isolated after three consecutive passages (7 days for each passage) on each cell line.

In Switzerland, in 2022, two fattening pig farms in the Canton of Vaud and the Canton of Fribourg observed an outbreak of disease with clinical neurological signs, including ataxia, nystagmus, lateral recumbency, and occasional fever, with reported on-farm mortality rates of 1% and 10% for the first and second farm, respectively. Animals from the second farm had been recently acquired from the first farm. Animals of the first farm were not treated. However, a few animals were reported to have spontaneously recovered a few days after the onset of neurological signs. Animals of the second farm were treated with Amoxicillin, with no improvement in clinical signs. Two diseased pigs from the first farm (pig CHE/1 and CHE/2) and one from the second farm (pig CHE/3) were referred for necropsy to the Institute of Animal Pathology of the University of Bern. At necropsy, aside from multifocal, moderate, subacute stomach ulcers in pig CHE/1 and CHE/2, gross lesions were not observed in other organs. Bacteriological cultures of brain tissues samples of all animals did not show bacterial growth. Routine serological analyses for antibodies directed against african swine fever, classical swine fever, and porcine reproductive and respiratory syndrome virus were negative for all animals. PCR analyses for pseudorabies virus, porcine echovirus, porcine sapelovirus, and porcine enterovirus were negative.

### Histology shows severe non-suppurative polioencephalomyelitis

**Fig 1:**
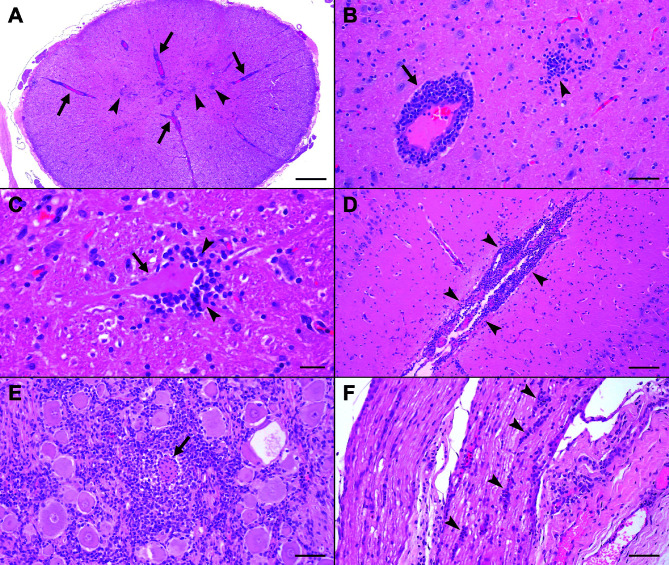
Representative histopathological lesions in Pig CHE/2 (**A, B, D–F**) and Pig CHE/1 (**C**), hematoxylin and eosin stain. (A) Spinal cord. Multifocal prominent perivascular cuffs (arrows) and glial nodules (arrowheads). (B) Thalamus. Higher magnification of a perivascular cuff (arrow) consisting predominantly of mononuclear cells, and of a glial nodule (arrowhead). (C) Medulla oblongata. Satellitosis/neuronophagia characterized by glial cells (arrowheads) surrounding and partially infiltrating a hypereosinophilic (necrotic) neuron (arrow). (D) Cerebral cortex. Lymphoplasmacytic meningitis (arrowheads). (E) Spinal ganglion. Multifocal infiltration of lymphocytes, macrophages, and fewer neutrophils surrounding and infiltrating a central necrotic neuron (arrow). (F) Spinal nerve. Lymphocytes, macrophages, and a few eosinophils infiltrate the nerve fascicles (arrowheads). Magnification: A = 2× (scalebar: 500 µm); B, E, F = 20× (scalebar: 50 µm); C = 40× (scalebar: 20 µm); D = 10× (scalebar: 100 µm).

Histologically, we observed lesions consistent with viral encephalomyelitis in the CNS of all four pigs. Intraparenchymal lesions were mild to severe and were more prominent in the gray than in the white matter. The lesions were distributed from the spinal cord to the cortex (Table 1). They consisted predominantly of multifocal mononuclear perivascular cuffs, glial nodules, gliosis, and mononuclear meningitis. Occasionally, we noticed perineuronal satellitosis/neuronophagia, neuronal necrosis, neuronal chromatolysis, axonal spheroids, and mild perivascular hemorrhages. We rarely found mononuclear ganglionitis and neuritis (Fig. 1). Additionally, pig CHE/3 showed mild, multifocal lymphoplasmacytic myocarditis, hepatitis, and pyelonephritis, and mild, multifocal, lymphoplasmacytic, and eosinophilic enterocolitis.

**TABLE 1:** Distribution and semi-quantitative assessment of histopathological lesion severity in the central nervous system of affected animals^*a*^

CNS region	Pig CHE/1	Pig CHE/2	Pig CHE/3	Pig AUS/1
Spinal cord	+++	+++	++	+++
Spinal ganglia	+	+	+	n.a.
Spinal nerves	+	+	+	+
Brainstem	+++	++	+	+++
Cerebellum	++	++	+	++
Midbrain	+++	++	+	+++
Hippocampus	−	−	+	n.a.
Thalamus	++	++	+	n.a.
Basal nuclei	++	++	++	+
Cerebral cortex	++	++	+	+++
Pineal gland	n.a.	n.a.	+	n.a.

^*a*^ +++: Severe lesions; ++: moderate lesions; +: mild lesions; −: no lesions; n.a.: not available.

### Discovery of porcine bastroviruses

Based on the histopathological lesion pattern indicating viral infection of the CNS and negative RT-qPCR tests for common neurotropic viral infections in pigs, we initiated an unbiased HTS and bioinformatics approach for virus identification and discovery in brain and spinal cord tissue samples of pig AUS/1 (conducted at the CSIRO Australian Centre for Disease Preparedness in 2015) and CHE/1 (conducted at the University of Bern, Switzerland in 2022) to determine the disease etiology. HTS of cDNA libraries prepared from total RNA extracts of a CNS tissue sample from pig AUS/1 and *de novo* assembly of the reads resulted in scaffolds ranging from 582 to 1,985 nt (in total covering 6,030 nt) with similarities to PoBastV (Table S1). There were a few sequence uncertainties, which were determined by RT-qPCR and Sanger sequencing. This resulted in a single contig sequence of 5,985 nt representing a novel near full-length BastV genome with the highest sequence similarity of ~82% to PoBastV_USA_2027-1 (GenBank MK387176). This strain was designated PoBastV AUS/2015 (GenBank accession number PP999617).

**Fig 2:**
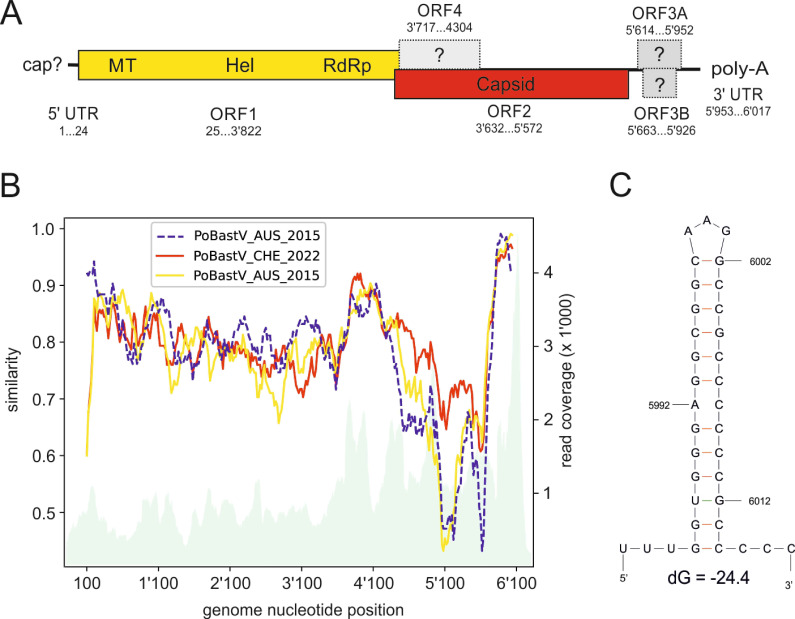
Molecular features of PoBastV AUS/2015 and CHE/2022. (A) Schematic representation of the genome organization. The range of the ORFs is indicated with their nucleotide position in the PoBastV-CH22 genome. Note that PoBastV AUS/2015 reveals the same genomic organization. Analysis with motif finder tool (https://www.genome.jp/tools/motif/; accessed 26 June 2023) revealed a viral methyltransferase, viral helicase 1, and RdRp2 domain in the ORF1, a capsid domain in ORF2, and a RHIM motif (RIP homotypic interaction motif) in ORF4, which has a function in regulation of apoptosis and immune modulation of cells (15, 16). The protein putatively encoded by ORFs 3A and 3B revealed no known protein motifs. Putative ORFs are indicated by question marks. (B) Pairwise comparison of nucleic acid sequence similarities (left y-axis) of PoBastV CHE/2022 and AUS/2015 (continuous lines) to the porcine bastrovirus reference strain (PoBastV Viet Nam/17489_85/2015; Genbank NC_032423) and of PoBastV AUS/2015 (dashed line) to CHE/2022. The gray shaded graph represents the coverage of the PoBastV CHE/2022 sequence by HTS reads (Integrative Genomics Viewer version 2.14.1). (C) Secondary structure prediction for the genomic 3′ UTR reveals a prominent stem-loop. Genome position of the nucleotides and the Gibbs free energy change (ΔG) are indicated.

In Switzerland, a similar HTS and bioinformatics approach resulted in hits to two virus database entries: a 610 nt scaffold for a porcine endogenous retrovirus and a 6,050 nt scaffold for a PoBastV (Table S1). While porcine endogenous retrovirus sequences are a common finding in HTS data in healthy pigs and are unrelated to diseases, it is different for the PoBastV. The precise genome termini of the PoBastV genome were determined by 5′ and 3′ rapid amplification of cDNA ends (RACE). The resulting full-genome sequence was comprised of 6,017 nt (Fig. 2A). Genetically, the closest relative virus was porcine PoBastV JPN/Iba-Ohata2/2015 (GenBank accession LC577872) with an average of 83.7% nt sequence identity and 95.5%–98.2% identity on the protein level (Fig. 2B; Table S1). This BastV strain was designated PoBastV CHE/2022 (GenBank accession number PP999616).

PoBastV CHE/2022 and AUS/2015 were 81% identical in their nucleic acid sequence. The genomic organization of both strains was like that of other BastVs, with ORF 1 encoding a protein with motifs indicating helicase, methyltransferase, and RdRp function and ORF 2 encoding a protein with motifs of the astrovirus capsid protein. In addition, in both viral sequences, we found a third ORF (ORF3A) of 336 nt, with an internal ORF of 264 nt (ORF3B) at the 3′ terminus of the genome. Yet another internal ORF (ORF4) of 588 nt in PoBastV CHE/2022 and 594 nt in PoBastV AUS/2015, respectively, overlapped with the 5′ end of ORF2 (Fig. 2A).

When we analyzed PoBastV sequences available on GenBank, we found that ORF3A, ORF3B, and ORF4 appear to be conserved in all of them. However, these ORFs have remained unannotated so far, most likely for ORF3A and 3B, because most of these sequences were incomplete at the 3′ terminus (Fig. S1 and Table S2). Sequence similarities were 84%–98% for ORF3A, 88%–98% for ORF3B, and 86%–97% for ORF4 (Table S3). ORF3A in PoBastV AUS/2015 and PoBastV CH/2022 does not overlap with the 3′ terminus of ORF2; however, this is different for all other PoBastV genome sequences, in which it overlaps with ORF2 by 47 nt (Fig. S1).

**Fig 3:**
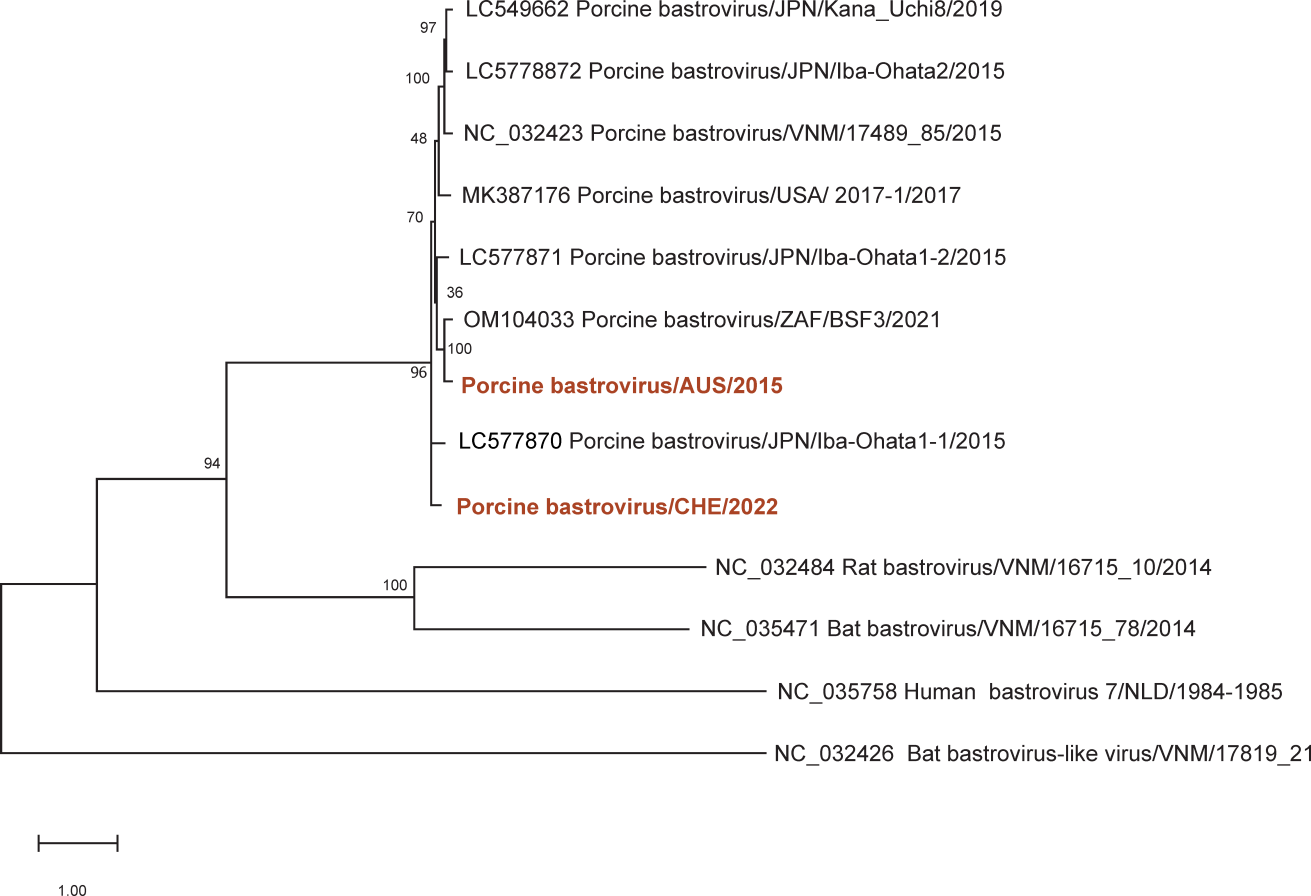
Phylogenetic comparison of PoBastV-CH/2022 and PoBastV AUS/2015 genome sequences with known PoBastV. The analysis was performed using near full-length PoBastV sequences (>5,000 nt) and reference sequences of human, rat, and bat BastV. GenBank accession numbers are presented together with the strain name and the year of detection. The alignment was performed using MAFFT (Ver. 7.475) software (17). A maximum-likelihood tree was constructed with IQ-Tree 2 (18) (Ver. 2.0.3) with 5,000 bootstrap replicates.

Analysis of the 5′ and 3′ UTR for secondary RNA structures *in silico* revealed a predicted prominent stem-loop of 29 nt within the 3′ UTR of both strains (Fig. 2C). Whether this structure is conserved in other bastroviruses is difficult to assess, because in most available sequences the 3′ UTRs are missing. Phylogenetically, both the Swiss and the Australian genome sequences clustered with PoBastV identified in feces samples of pigs from Japan, Vietnam, and the USA (9, 14) (Fig. 3).

### Porcine bastroviruses AUS/2015 and CHE/2022 are neurotropic

**Fig 4:**
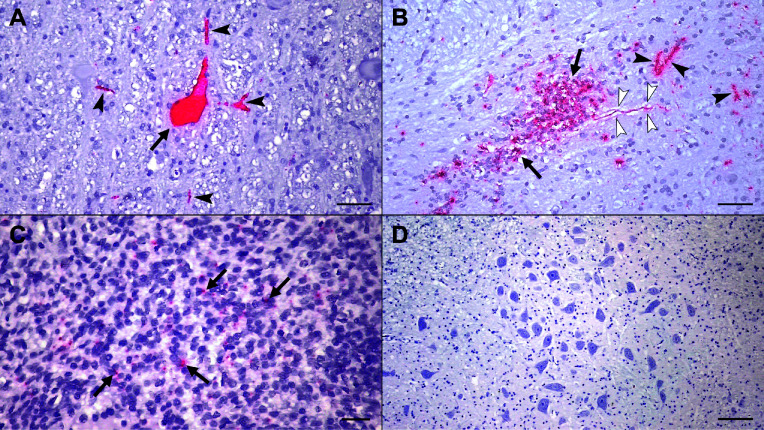
*In situ* hybridization (ISH) staining for PoBastV CHE/2022 (**A, C**) and AUS/2015 (**B**) RNA in FFPE tissues. (A) Medulla oblongata (Pig CHE/2). Viral RNA (red) within a neuronal cell body (arrow) and neuronal processes (arrowheads). (B) Medulla oblongata (Pig AUS/1). Viral RNA in association with confluent glial nodules (arrows), neuronal processes (black arrowheads), and an axon (white arrowheads). (C) Mesenteric lymph node (Pig CHE/3). Viral RNA in association with lymphoid cells (arrows). (D) Negative control for ISH. Neurons, neuropil, and glial cells in the medulla oblongata of a pig without inflammatory lesions display no viral RNA. Magnification: A = 20× (scalebar: 50 µm); B, C = 40× (scalebar: 20 µm); D = 10× (scalebar: 100 µm).

To further assess the association of PoBastV CHE/2022 and AUS/2015 infections and the observed neuropathological lesions, we performed RNA *in situ* hybridization (ISH) with customized probes targeting the ORF2. In all four cases, viral RNA was found in neurons and their processes, reflecting viral neurotropism (Fig. 4, Fig. S2 and S3). While relatively few cells were stained in pigs CHE/1 to CHE/3, pig AUS/1 showed more frequent and intense staining throughout CNS sections (Table 2). We also found RNA labeling in axons and within ganglia. Furthermore, viral RNA was found in the mesenteric lymph nodes of pigs CHE/1, CHE/2, and CHE/3, but not in other extra-neural tissues. RT-qPCR revealed high amounts of PoBastV RNA in CNS samples (cq values between 17 and 22) and a whole-blood sample (cq value of ~22), and relatively low amounts of viral RNA in peripheral organs (cq values between 29 and 32) of pig AUS/1 (Table S2). Overall, these findings suggest that these PoBastV strains are not only neurotropic but may also cause viremia and systemic infections.

**TABLE 2:** Distribution and ISH labeling intensity of PoBastV RNA in affected piglets^*k*^

Anatomical site	Pig CHE/1	Pig CHE/2	Pig CHE/3	Pig AUS/1
Spinal cord	+^*b*^^,*c*,*e*^, ++^*a*^	+^*b*^, +++^*a*^^,*c*,*d*,*e*^	+^*e*^	++^*c*^, +++^*a*^^,*b*,*d*,*e*^
Spinal ganglion	−	+^*f*^	−	n.a.
Spinal nerves	−	−	−	+^*e*^, ++^*b*^
Brainstem	+^*c*^^,*e*^, +++^*a*^^,*d*^	+^*a*^^,*c*,*e*^	−	++^*c*^, +++^*a*^^,*b*,*d*,*e*^
Cerebellum	−	+^*a*^^,*e*,*f*,*g*^	−	+^*a*^, ++^*e*^^,*f*,*g*^
Midbrain	+^*c*^^,*e*^	+^*c*^^,*e*^	n.i.	+^*b*^^,*d*^, +++^*a*^^,*d*,*e*^
Hippocampus	n.i.	−	n.i.	n.a.
Basal nuclei	n.i.	n.i.	−	++^*b*^^,^^*c*^^,^^*d*^
Cerebral cortex	−	n.i.	−	++^*a*^^.*c*,*d*,*e*^
Mesenteric lymph nodes	+^*h*^	+^*h*^^,*i*^	+*^i^*	−
Tonsil	−	−	−	n.a.
Stomach^*j*^	n.a.	n.a.	n.a.	−
Small intestine^*j*^	−	−	−	−
Large intestine^*j*^	n.a.	n.a.	−	−
Heart	n.a.	n.i.	−	−
Lung	n.a.	n.a.	−	−
Liver	n.a.	n.a.	−	−
Kidney	n.a.	n.a.	−	−
Spleen	n.a.	n.a.	−	−

^*a*^ Neurons.

^*b*^ Axons.

^*c*^ Neuropil.

^*d*^ Neuronal processes.

^*e*^ Glial nodules, myelin sheath.

^*f*^ Intragangliar nerve fibers, Purkinje cells.

^*g*^ Dendritic arbors.

^*h*^ Lymphoid cells in trabecular sinus.

^*i*^ Lymphoid cells in subcapsular sinus.

^*j*^ Including associated nervous plexuses.

^*k*^ +++: Abundant signal, ++: moderate signal, +: rare signal, −: no signal, n.i.: not included (in the analyzed section), n.a.: not available.

### Porcine bastrovirus CHE/2022 replicates in porcine intestinal cells

**Fig 5:**
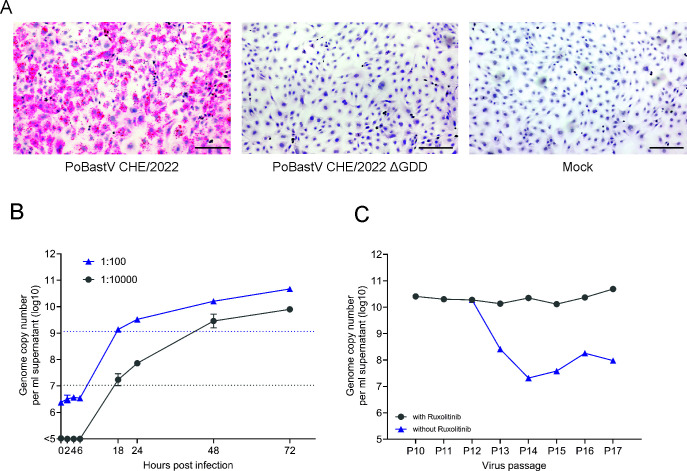
Rescue of PoBastV CHE/2022 by reverse genetics. (A) RNA *in situ* hybridization of IPEC-J2 cells infected with PoBastV CHE/2022 (supernatant passage 10 after rescue). Red dots show labeling of viral RNA within the IPEC-J2 cells, but not in cells infected with supernatant of cells that were transfected with the PoBastV-CH22 ΔGDD mutant and in mock-infected cells. Scale bar = 100 µm, magnification = 10×. (B) PoBastV CHE/2022 growth kinetics in IPEC-J2 cells. Supernatant of cells infected with two different dilutions (1:100, blue triangles; 1:10,000, gray dots) of the P10 virus stock was collected at the indicated time points post-infections and tested by RT-qPCR. (C) Effect of Ruxolitinib on PoBastV CHE/2022 replication. Supernatants were passaged on IPEC-J2 cells with (gray dots) and without (blue triangles) Ruxolitinib and tested by RT-qPCR for viral copy numbers.

Because suitable material of PoBastV CHE/2022 and AUS/2015 for cell culture inoculation was no longer available, we aimed at rescuing the virus by reverse genetics. We constructed a molecular cDNA clone of PoBastV CHE/2022 (pEX_PoBAstV-CH/2022) together with a replication-deficient mutant in which the GDD motif of the RNA-dependent RNA polymerase was deleted (pEX_PoBastV-CHE/2022 ΔGDD), transcribed viral genomic RNAs *in vitro,* and transfected the RNA into swine kidney SK-6 cells. Supernatant of transfected cells was then used to infect SK-6, PK-15, IPEC-1, and IPEC-J2 cells. Supernatants of these cells were further passaged ten times with and without the addition of Ruxolitinib, a Janus-kinase inhibitor, on the respective cell lines. As expected, viral RNA was not detected by ISH in cells infected with supernatants of SK6 cells transfected with the replication-deficient PoBastV CHE/2022 ΔGDD mutant RNA. When we transfected wild-type PoBastV CHE/2022 viral RNA, the ISH was negative at passage 10 (P10) in SK-6, PK-15, and IPEC-1 cells with or without Ruxolitinib. IPEC-J2 cells not treated with Ruxolitinib were also ISH negative at P10. However, we found a strong ISH signal at P10 in Ruxolitinib-treated IPEC-J2 cells, indicating that infectious PoBastV was rescued and passaged (Fig. 5A). The infectious titer of the P10 supernatant was determined to be 2.26 × 10^7^ TCID_50_/mL in IPEC-J2 cells. To demonstrate the viral replication kinetics, we infected IPEC-J2 cells with different amounts of PoBastV CHE/2022 P10 supernatant and determined viral RNA levels at different time points post-infection by qRT-PCR. We observed an exponential increase in viral RNA levels of at least four orders of magnitude, with a plateau phase starting at ~24 h post-infection (Fig. 5B). HTS of total RNA extracted from P10 supernatants of IPEC-J2 cells and reference-guided read-assembly of HTS reads resulted in a full-length consensus sequence with 100% identity to the PoBastV CHE/2022 sequence (data not shown). Because virus rescue in IPEC-J2 cells appeared to be dependent on the presence of Ruxolitinib, we further aimed to investigate whether Ruxolitinib was necessary for sustainable virus propagation. PoBastV CHE/2022 of P10 supernatant was further passaged twice in IPEC-J2 (P11 to P12) with the addition of Ruxolitinib. Then the virus was further passaged with and without Ruxolitinib (P13 to P17). While the level of viral RNA remained stable at >10^10^ copies/mL in the presence of Ruxolitinib, removal of Ruxolitinib resulted in its reduction to ~10^8^ copies/mL, indicating that it is not essential for PoBastV CH/2022 propagation in IPEC-J2 cells (Fig. 5C).

**Fig 6:**
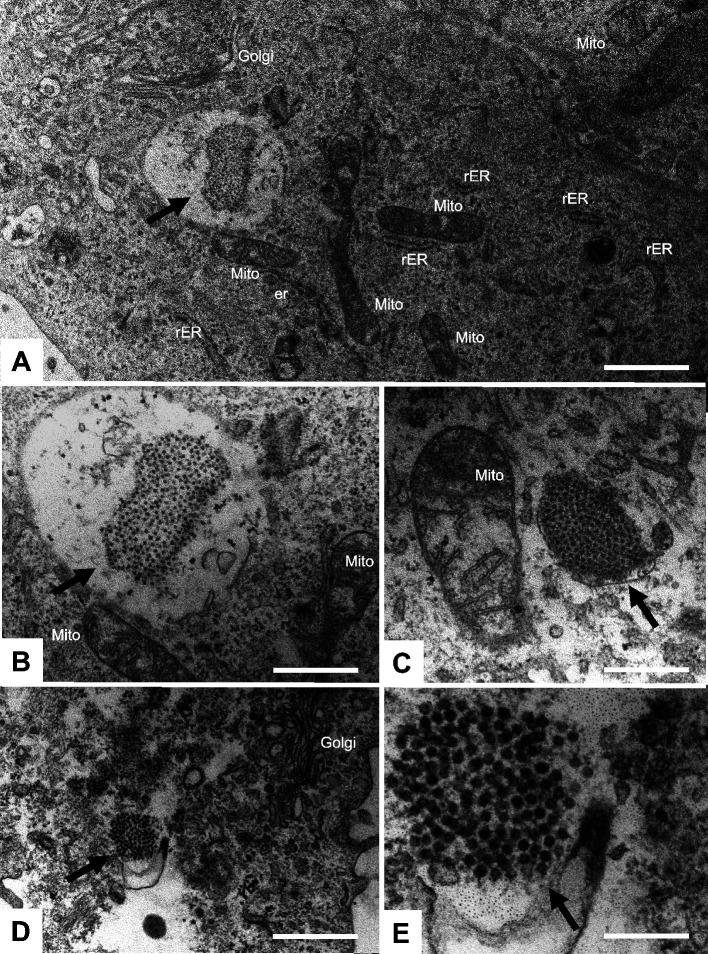
Transmission electron microscopy of IPEC-J2 cells infected with PoBastV CHE/2022. A–C were fixed and processed at 12 h p.i., D and E at 24 h p.i. (A) Low magnification view of the cytoplasm of an infected cell with the paracrystalline arrays composed of putative PoBastV virions (arrow). (B) The compartment with virus particles is shown at higher magnification. (C) A view onto a paracrystalline array (arrow) located alongside a mitochondrion (mito). (D) An infected cell fixed at 24 h p.i., and the compartment with putative virions (indicated by the arrow) is shown at higher magnification in (E). Bars in A = 1.2 µm, B = 0.7 µm, C = 0.25 µm, D = 0.84 µm, and E = 0.22 µm. Mito = mitochondrion, rER = rough endoplasmic reticulum, Golgi = Golgi apparatus.

To confirm the presence of PoBastV CHE/2022 viral particles in IPEC-J2 cells, we performed ultrastructural analysis by transmission electron microscopy and demonstrated membrane-associated and intracytoplasmic paracrystalline arrays of virus-like particles as well as individual particles of 27–30 nm diameter, which likely correspond to PoBastV virions (Fig. 6). Collectively, these data provide evidence that infectious PoBastV CHE/2022 can be rescued from a molecular clone and replicates efficiently in an intestinal porcine epithelial cell line.

## DISCUSSION

BastVs have only recently been described, and their pathogenicity is poorly understood. Here, we provide the first evidence that they can invade the nervous system and are associated with a severe neurological disease in pigs. The observed non-suppurative polioencephalomyelitis is typical of viral infections. We demonstrated the presence of PoBastV RNA in spinal cord and brain tissues of affected animals in association with these lesions. This observation and the absence of other neuroinfectious agents, demonstrated by conventional molecular diagnostics and viral metatranscriptomics, plausibly support a causal relationship between PoBastV neuroinfection and disease (19). Additional samples from animals of the affected farms were not available; however, the case history suggests that PoBastV infections may lead to mortality rates of up to 10%. Further disease surveillance and diagnostic targeting of PoBastV in pig farms with cases of neurological disease are required to assess disease prevalence and geographic occurrence.

To date, PoBastV has been detected only in feces and saliva of pigs (9, 14, 20). In addition to CNS infection, we found evidence for the presence of PoBastV RNA in the mesenteric lymph nodes and in the blood of affected animals. However, intestinal tissue samples were free of relevant histopathological lesions and negative for PoBastV by ISH. In only the Australian case, the peripheral organs were positive for PoBastV RNA by RT-qPCR. Viral RNA loads in these organs were lower compared to those in the blood, which might indicate that organ positivity was due to residual blood. However, CNS tissues revealed viral RNA levels higher than in the blood, suggesting that the virus was indeed replicating in the CNS. Although these findings raise the possibility of viremia and viral spread from the intestine to the nervous system, the disease pathogenesis and route of neuroinvasion remain unknown and should be addressed in future research.

Both PoBastV strains identified in this study are genetically similar to each other and to previously described PoBastV detected in feces samples. Besides ORF1 and ORF2, we found two additional 3′-terminal ORFs (3A and 3B), of which the functionality remains to be established. Analysis of the genomic sequences of PoBastV available on GenBank revealed similar ORFs; however, they have remained unannotated so far. Putative open reading frames partially overlapping with the 3′ end of ORF2 have also been identified in BastVs sequences in human and rat feces (1, 21). Contrastingly, ORF4, which overlaps with ORF2, has not yet been described, although it can be found in all PoBastV sequences on GenBank. A similar ORF is present in some astroviruses and in HEV, termed ORFX and ORF3, respectively. For human astrovirus 1, ORFX is proposed to encode a viroporin-like protein that facilitates the egress of viral particles from infected cells (22). In HEV, the encoded protein is proposed to have a function in the interaction with the innate immune system and in viral particle release (23). Whether the protein encoded by ORF4 in PoBastVs reveals similar functions remains to be investigated. Another similarity to astroviruses is the predicted stem-loop RNA structure at the genomic 3′ terminus. In human astroviruses and coronaviruses, a conserved secondary structure, the stem-loop-2-motif (s2m) (24), is indispensable for astroviral RNA replication (25). We therefore speculate that the 3′ terminal stem-loop motif in PoBastVs performs a similar function, although the sequence is not identical to that of the s2m.

The molecular biology of BastV has been poorly investigated so far, mainly due to the lack of virus isolation and cell culture propagation. By swiftly constructing a molecular clone and identifying a permissive porcine intestinal cell line, we were able to overcome this limitation, i.e., to rescue PoBastV in SK6 cells and to further propagate it in IPEC-J2 cells.

Not only can we now leverage this system to genetically engineer the virus and to explore the function of viral proteins and RNA structures, but we can also perform experimentally controlled infection studies in pigs to better understand pathogenesis and neuroinvasion mechanisms. IPEC-J2 are permanent, non-transformed porcine enterocytes originally isolated from the small intestine of piglets; thus, they are likely natural targets of PoBastV. However, efficient viral replication of PoBAstV CHE/2022 required suppression of innate immunity, suggesting the importance for the virus to overcome the cellular antiviral defense. As IPEC-J2 cells show growth characteristics similar to enterocytes *in situ*, i.e., formation of microvilli at the apical side and polarization (26), they may be a valuable model to investigate aspects of viral entry and host invasion of PoBastV *in vitro*.

In conclusion, our findings suggest a causative relation between BastV and fatal neurological disease in domestic pigs. Further research is needed to determine transmission routes, disease burden, geographic occurrence, and other factors. However, with the ability to culture the PoBastV in porcine intestinal cells and to genetically engineer the virus, research into fundamental aspects of the molecular biology of BastV is now possible.

## MATERIALS AND METHODS

### Pathology

Samples from three pigs from Switzerland (pigs CHE/1 to CHE/3) and one pig from Australia (pig AUS/1) were assessed. At necropsy, brain, spinal cord, mesenteric lymph nodes (all pigs), small intestine and tonsils (pigs CHE/1, CHE/2, and CHE/3), large intestine, lung, liver, spleen, and kidney (pigs AUS/1 and CHE/3) samples were collected, fixed in 4% neutral buffered formalin, embedded in paraffin, processed routinely for histopathological examination, and stained with hematoxylin and eosin. Unfixed brain (pig AUS/1) and spinal cord of pig CHE/1 samples were collected and stored at −20°C for further examinations. This study comprised exclusively the use of animal tissues submitted for pathologic diagnostic purposes and does not require ethical approval by an institutional board.

### High-throughput sequencing and bioinformatics

Details on the RNA extraction procedures, HTS cDNA library preparations, and the bioinformatics tools can be found in the supplemental material.

### Rapid amplification of cDNA ends

5′ and 3′ terminal genome sequences were determined by RACE using the 5′ and 3′ RACE System for Rapid Amplification of cDNA Ends, Version 2.0 (Invitrogen). RT-PCR reactions were conducted using the GoTaq Green MasterMix (Promega), and products were Sanger-sequenced with the corresponding gene-specific primers. The sequence information for the primers is available in Table S3.

### Quantitative reverse transcription-PCR

Two different RT-qPCR protocols were developed, for PoBastV AUS/2015 and PoBastV CHE/2022, respectively. Details on these protocols are provided in the supplemental material.

### Molecular cDNA clone of PoBastV CHE/2022

A molecular cDNA clone of the PoBastV CHE/2022 genome, flanked by a T7 promoter upstream of the 5′ UTR and a NotI restriction site downstream of the poly-A tail (56 adenine bases), was synthesized at Eurofins Genomics (Germany). The construct was cloned in a pEX cloning vector, resulting in pEX_PoBAstV-CH/2022. The T7 promoter included two additional guanine bases upstream of the authentic guanine at the 5′ UTR of the viral genome. The NotI restriction site was used to linearize the plasmid prior to *in vitro* RNA transcription. As a control, a construct (pEX_PoBastV-CHE/2022 ΔGDD) was designed in which the RNA-dependent RNA-polymerase was knocked out by deleting the GDD motif (encoded by positions 3,229–3,237 of the genome), using the In-Fusion HD Cloning Kit (Takara), resulting in a replication-incompetent viral genome. Primer sequences are available in Table S3.

### *In vitro* RNA transcription

Plasmids were amplified in Stellar competent *E. coli* (Takara Bio) and purified using the PureYield Plasmid Midiprep System (Promega), according to the manufacturer’s instructions. After linearization of 10 µg plasmid with NotI (0.5 U/µL; ThermoFisher), the DNA was purified with 25:24:1 (vol/vol/vol) phenol, chloroform, isoamyl alcohol (Sigma-Aldrich), followed by a chloroform washing step and an ethanol sodium acetate precipitation. Finally, the sample was washed once with 75% vol/vol ethanol. The RNA was transcribed with the mMESSAGE mMACHINE T7 Transcription Kit (Invitrogen) using 1 µg of purified DNA, according to the manufacturer’s instructions.

### Cell culture

Swine kidney cells (SK-6) (27) and porcine kidney cells (PK-15) (28) were maintained at 37°C and 5% CO_2_ in DMEM, high glucose, pyruvate (Gibco), supplemented with 10% fetal calf serum, and 1% penicillin/streptomycin. IPEC-1 and IPEC-J2 (29) were also maintained at 37°C, 5% CO_2_, but in DMEM/F-12, GlutaMAX supplement (Gibco), and supplemented with 10% fetal calf serum and 1% penicillin/streptomycin. Cells were transfected or infected at 80%–90% confluency.

### Virus rescue

SK-6 cells were seeded in a 6-well plate and transfected with 2.5 µg of *in vitro* transcribed PoBastV RNA using the TransIT-mRNA Transfection Kit (Mirus Bio) when they reached a confluency of ~80%. After 8 h of incubation, the medium was changed, and the cells were incubated for an additional 72 h at 37°C. Then, the supernatant was collected and directly frozen.

### Virus passaging

One day before infection, SK-6, PK-15, IPEC-1, or IPEC-J2 cells were seeded at a density of approximately 700,000 cells/well in 6-well plates, 90,000 cells/well in 24-well plates, or 28,000 cells/well in 96-well plates. When cells reached around 80% confluency, they were treated with 1 µM Ruxolitinib (Med Chem Express) for 1 h before infection or remained untreated. The cells were then washed with PBS and infected with supernatant from transfected SK-6 cells for 90 min. After that, the cells were washed again, and Ruxolitinib (1 µM) was added to the medium. The cells were incubated for 72 h at 37°C, 5% CO_2_. Then, supernatants were harvested, centrifuged at 300 × *g* for 10 min, and stored at −80°C until further passaging.

### *In situ* hybridization

RNA *in situ* hybridization was conducted to detect viral RNA in formalin-fixed paraffin-embedded (FFPE) tissue sections and infected cell cultures. We used the RNAscope technology (2.5 HD Assay-RED; ACDbio) with probes targeting the nucleotide positions 4,299–5,240 of the PoBAstV-CHE/2022 (Cat no. 1198181-C1, 20ZZ probes) and the nucleotide positions 3,684–4,543 of the PoBastV-AUS/2015 (Cat no. 1302171-C1, 20ZZ probes) genomes, respectively, with the following modification: incubation with the reagent Amp 5 of the kit was extended to 1 h instead of 30 min in FFPE samples (but not for cultured cells), to increase signal intensity. To detect the viral RNA in cell culture, cells were seeded in eight-well Nunc Lab-Tek Chamber Slide systems (Merck). At 80%–90% confluency, they were infected with 100 µL of supernatant of PoBAstV-CH22-infected cells. The cells were incubated for 24 h (SK6, PK15 IPEC-J1) or 8 h (IPEC-J2) and then fixed with 10% neutral buffered formalin. Microphotographs were taken using an Eclipse E600 microscope (Nikon, Minato, Japan) and an Axiocam 208 color camera (Zeiss). The images were processed using ZEN (blue edition; Zeiss).

### Virus titration

Supernatant of passage PoBastV on IPEC-J2 cells was 10-fold serially diluted, and each dilution was inoculated into the IPEC-J2 cells in 96-well culture plates in six replicates per dilution. The infection procedure was the same as described above, using medium containing Ruxolitinib. After 72 h, supernatants were harvested and tested by RT-qPCR. A replicate was considered positive when the Cq value was ≤40. The TCID_50_ was calculated according to the Spearman–Kärber method using an online tool (https://www.virosin.org/tcid50/TCID50.html).

### Virus kinetics

IPEC-J2 cells were seeded in 24-well plates and incubated overnight. The next day, cells were infected with passage 10 PoBastV CHE/2022 at two different dilutions: 1:100 and 1:10,000 (corresponding to a multiplicity of infection). The infection experiment was the same as described earlier, including Ruxolitinib. Supernatant was collected at multiple time points: 0, 2, 4, 6, 18, 24, 48, and 72 h post-infection. Viral RNA was extracted from the collected supernatants, and RT-qPCR was performed as described above.

### Transmission electron microscopy

Sample processing for transmission electron microscopy was essentially performed as described previously (30). Details are provided in the supplemental material.

## Data Availability

Genome sequences of PoBastV CHE/2022 (https://www.ncbi.nlm.nih.gov/nuccore/PP999616) and PoBastV AUS/2015 (https://www.ncbi.nlm.nih.gov/nuccore/PP999617) are available from the National Center for Biotechnology Information (NCBI) GenBank. HTS reads are available from the NCBI sequence read archive (Bioproject accession number PRJNA1284599).

## SUPPLEMENTAL MATERIAL

Supplemental material jvi.01130-25-s0001.pdf Figures S1 to S4, supplemental methods, and Tables S1 to S5.

ASM does not own the copyrights to Supplemental Material that may be linked to, or accessed through, an article. The authors have granted ASM a non-exclusive, world-wide license to publish the Supplemental Material files. Please contact the corresponding author directly for reuse.
