# Supplementary material for: Discovery and rescue of porcine bastroviruses associated with polioencephalomyelitis in domestic pigs
Source: J Virol. 2025 Aug 18;99(9):e01130-25. doi: 10.1128/jvi.01130-25 (PMC12456002; doi:10.1128/jvi.01130-25)
Supplement: Supplemental material — Figures S1 to S4, supplemental methods, and Tables S1 to S5. [file jvi.01130-25-s0001.pdf]

Supplementary material

Supplementary

Figures:

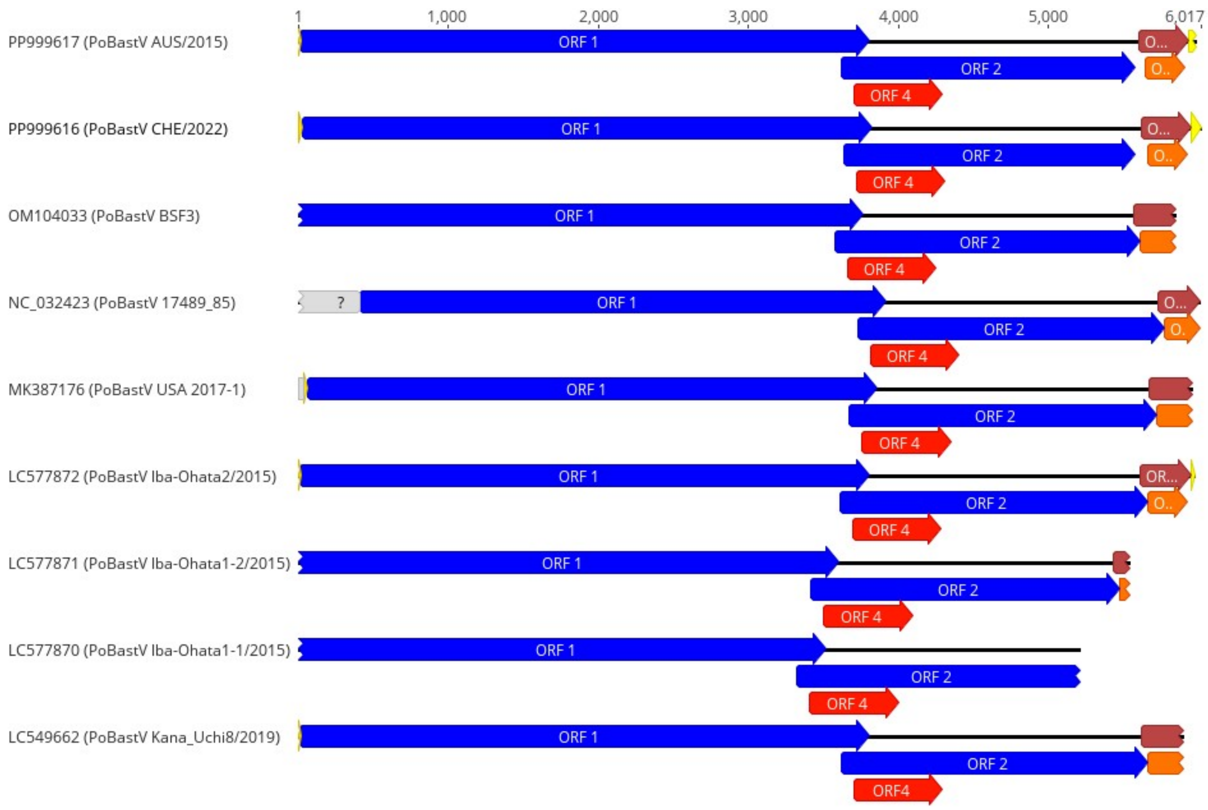

Figure S1: Comparison of the genome organisation of PoBastV. GenBank accession numbers are indicated. Sequences were analysed for the presence of open-reading frames (ORFs) and UTRs (yellow arrows) using Geneious Prime v. 2025.0.3. ORF and ORF 2 (blue arrows) were annotated in GenBank entries, while ORF 3A (brown) ORF 3B (orange) and ORF 4 (red) were not. Ragged ends indicate truncated ORFs,

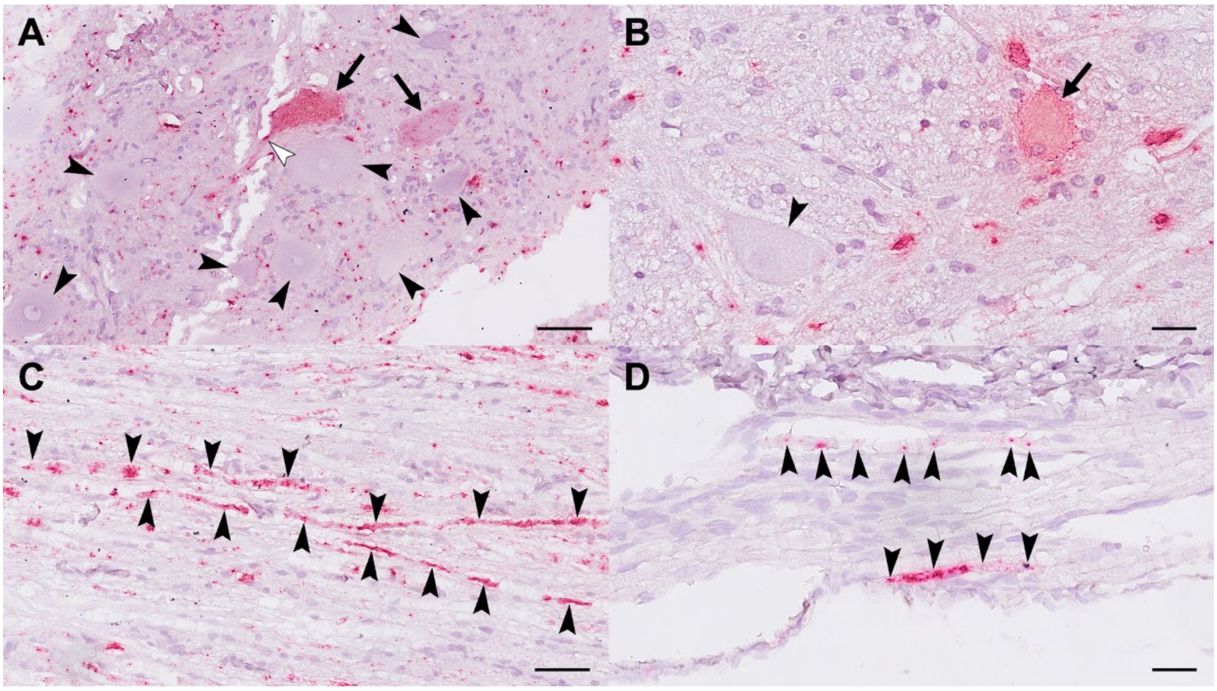

Figure S2: In situ hybridization staining for PoBastV AUS/2015 RNA in neurons and neuronal processes of pig AUS/1. A, B: Viral RNA (red) within neuronal cell bodies in the spinal cord (A) and medulla oblongata (B) (arrows) adjacent to neurons displaying no viral RNA (black arrowheads); in one neuron, the positive signal extends into the axon hillock (A, white arrowhead). C, D: Viral RNA within axons in the spinal cord (C) and a spinal nerve (D) (arrowheads). A, C=20x (scalebar: 50 µm); B, D=40x (scalebar: 20 µm).

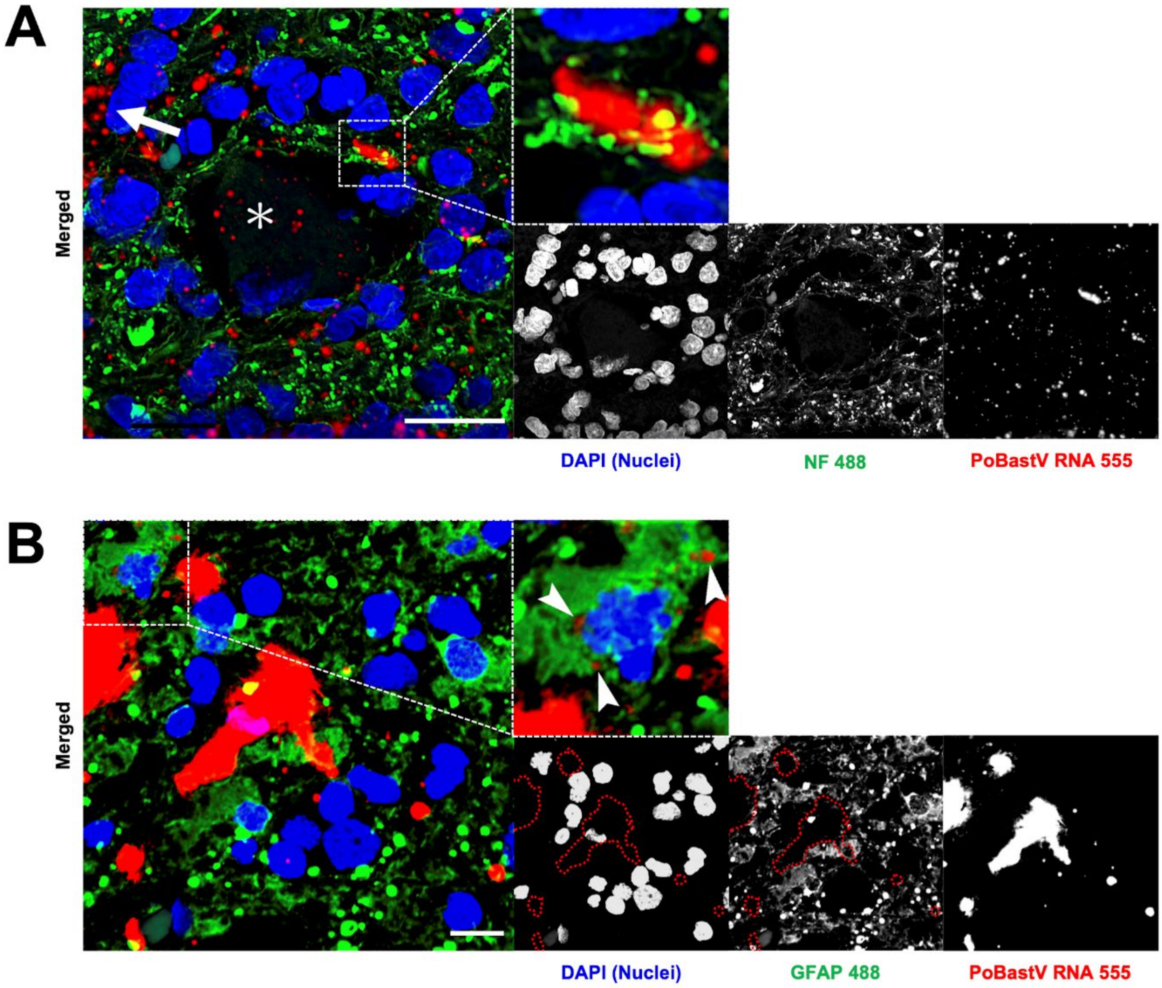

Figure S3: Detection of PoBastV AUS/2015 (A) and CHE/2022 (B) RNA through fluorescent *in situ* hybridization (FISH, red) in the medulla oblongata of pig AUS/1 (A) and the spinal cord of Pig CHE/2 (B). A) Viral RNA signal is present in the soma of a neuron (asterisk), as well as in association with phagocytes surrounding this neuron (arrow) and with Neurofilament (NF, green)-immunopositive neuronal processes (inset). B) Viral RNA signal (red dotted lines) does not preferentially overlap with GFAP immunolabeling (green); only few, small foci of viral RNA are observed in association with an astrocyte (inset, arrowheads). A=60x, 2.12 zoom (scalebar: 50 µm); B=60x, 2.68 zoom (scalebar: 10 µm).

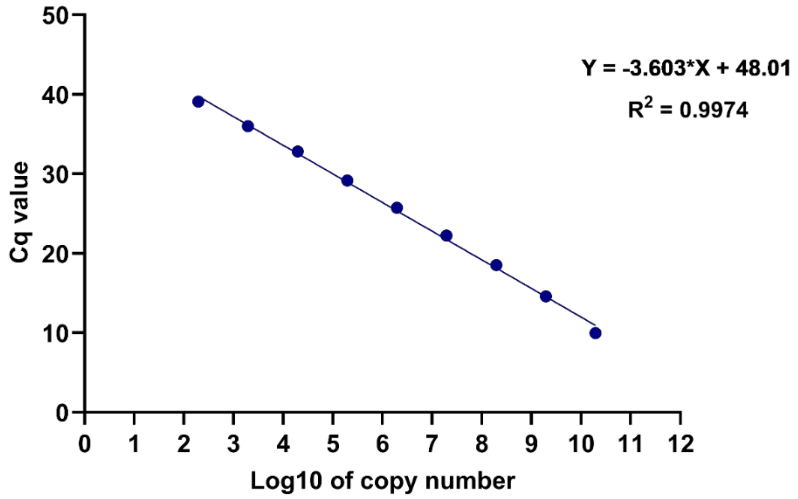

Figure S4: Standard curve for the PoBastV CHE/2022 RT-qPCR: RNA was in vitro transcribed from pEX-PoBastV CHE/2022 tested in 10-fold dilutions. Mean values of three independent experiments are shown.

Supplementary Materials and Methods

High-throughput sequencing (HTS) and bioinformatics

For pig AUS/1, total RNA was extracted from supernatant of 10% tissue homogenates of pooled brain and spinal cord using the MagMax 96 Viral RNA Kit (ThermoFisher Scientific) in a MagMAX Express Magnetic Particle Processor (ThermoFisher Scientific) following manufacturer's instructions. The RNA was treated with the Ribo-Zero rRNA Removal Kit (Illumina) to remove rRNA from total RNA. A HTS cDNA library was prepared with the TrueSeq RNA Library Prep Kit, (Illumina, USA) per manufacturer's instructions. The library was then sequenced using the MiSeq Reagent Kit V2 (paired-end sequencing, 2x 150bp) on a MiSeq platform (Illumina). The HTS sequence data were analysed using the CLC Genomic WorkBench (v20) with standard parameters. The quality trimmed reads were the aligned to the pig reference genome (Sscrofa 10.2/susScr3). De novo assembly was conducted with the unmapped sequence reads to generate longer sequence contigs. The resultant sequences were analysed using the NCBI nucleotide database (BlastN) (v 2.14), and protein database (BlastX) by using Diamond (v. 2.1.8), respectively (1).

For pig CHE/1 RNA was extracted from a fresh spinal cord sample using 1 ml TRI Reagent® (Sigma Aldrich) per manufacturer instructions. Total RNA was then used to prepare HTS cDNA libraries (Corall total RNA-Seq kit, Lexogen), and sequenced on an Illumina NovaSeq 6000 machine in single-end mode (1x100 bp). After sequencing, the reads were quality filtered and trimmed (fastp v. 0.12.5, (2)). Reads were then mapped to the pig reference genome (Sus scrofa 10.1) with STAR v. 2.7.3a (3) and the unmapped reads were assembled with SPAdes v. 3.12.0 (4). The resulting scaffolds were screened for homologies by BlastN v. 2.7.1+ against viral reference sequence database in GenBank and by using the BlastX function in DIAMOND v. 0.9.18 (1) against the viral amino acid sequences in the non-redundant protein database in GenBank (https://www.ncbi.nlm.nih.gov/genbank/), respectively. Databases were downloaded on 19th February 2023. Scaffolds resulting in viral hits were further analysed in Geneious prime (v. 2023.2). Coverage plots for the BastV genome from pig CHE/1 were generated using bowtie2 (5) by remapping of the reads against the viral scaffold. Similarity plots were calculated and displayed with SimPlot++ (6) with a sliding window of 200 nt and 20 nt steps. RNA secondary structures were predicted with Mfold (7).

Double staining of PoBastV and Neurofilament/GFAP

Fluorescent ISH was performed using the RNAscope system (Advanced Cell Diagonostic, Netwark, NJ). The AUS-PoBastV probe was applied to the FFPE slide from pig AUS/1 while CHE-PoBastV probe was used for the FFPE slide from pig CHE/2. The RNAscope 2.5 Detection Kit-Red (Advanced Cell Diagnostics, Newark, NJ) was used according to the manufacturer's instructions with a modified protease pretreatment incubation time of 20 min to maintain tissue quality. Then, immunofluorescence (IF) was performed under humid conditions with different cell markers. Slides were washed in PBS-T and incubated with 10% normal goat serum for 20 min at room temperature. Then, one primary antibody was applied per experiment. Antibodies used were monoclonal mouse anti-human neurofilaments clone 2F11 (Dako Denmark A/S, Glostrup, Denmark), and polyclonal rabbit anti glial fibrillary acidic protein (GFAP) (Dako Denmark A/S, Glostrup, Denmark) antibodies. Neurofilament staining was incubated at 4°C overnight with a 1:100 dilution in PBS-T. GFAP staining was added for 1 h at 37°C with a 1:1000 dilution in PBS-T. After washing with PBS-T, secondary Alexa Fluor 488 goat anti-rabbit or goat anti-mouse antibody (Abcam plc, Cambridge, UK) was

applied at a 1:1000 dilution in PBS-T, together with DAPI BioChemica (AppliChem GmbH, Darmstadt, Germany) at a 1:10,000 dilution. The slides were incubated for 1 h at room temperature, then washed with PBS-T and distilled water, before being mounted with Glycergel®, Aqueous Mounting Medium (Dako Denmark A/S, Glostrup, Denmark). Correlation analyses were performed using an Olympus Fluoview FV3000 Confocal Laser Scanning Microscope (Olympus Europa, Hamburg, Germany).

RT-qPCR

For PoBastV AUS/2015 RNA was extracted from 4-µM sections of FFPE tissues, using the RNeasy FFPE Kit (QIAGEN). The blood sample was extracted with the QIAamp RNA Blood Mini Kit (Qiagen). Primers were designed based on the HTS results with Primer Express 3 (Applied Biosystems) (Table S3). The RT-PCR was performed in 96-well plates in a 25-µL reaction volume containing 5 µL of RNA, 12.5 µL of AgPath One-step RT-PCR buffer (Ambion), 1 µL of 25 × reverse transcriptase, 1.25 µL of 18 µM each primer, 1.25 µL of 5 µM probe, and 2.75 µL of nuclease-free water. The RT-qPCR assays were performed under the following conditions: 10 min at 45 °C for reverse transcription of RNA, 10 min at 95 °C, followed by 45 cycles of 95 °C for 15 s, 60 °C for 45 s using a 7500 Real-time PCR system (Applied Biosystems).

For PoAstV CHE/2022 RNA was extracted from cell-free supernatant of IPEC-J2 cells infected with PoBastV, using QIAamp Viral RNA Mini Kit (Quiagen). The primers and probe were designed using Geneious Prime (Biomatters). Reverse transcription was performed using the GoScript™ Reverse Transcription Kit (Promega) and the PoBastV CHE/2022 specific reverse primer. The synthesized cDNA was subsequently amplified and quantified by qPCR using the GoTaq® Probe qPCR Master Mix (Promega) and the following settings: 95°C for 2 minutes, 40 x (95°C for 3 sec., 60°C for 30 sec.). A standard curve was determined by testing 10-fold serial dilution of *in vitro* transcribed PoBastV CHE/2022 RNA in triplicates. The mean of the Cq values for each dilution was used for further calculations. The RNA copy number in the undiluted RNA extract was calculated based on the estimated molecular mass of the transcript using an online tool (https://endmemo.com/bio/dnacopynum.php, accessed 31st March 2025), resulting in 1.97 x 10^{11} genome copies/µl. The detection limit of the RT-qPCR was 1.97 x 10^{2} genome copies.

Transmission electron microscopy

Infected cells grown in 6-well plates were washed once in 100 mM sodium cacodylate buffer pH 7.3 and were fixed in cacodylate buffer containing 2.5% glutaraldehyde for 2 h at RT. Subsequently, cells were scraped, centrifuged into a pellet (10'000 ×g, 10 min, RT), washed twice in cacodylate buffer, and post-fixated in 2% osmium tetroxide in cacodylate buffer for 2 h. Following several washes in water, specimens were dehydrated in an ascending ethanol series (30–50–70–90% and three times 100%),and embedded in Epon 812 resin. Resin was polymerised at 65°C overnight. The block was trimmed, and 80-nm thick sections were cut using an ultramicrotome (Reichert Jung), loaded onto 200-mesh copper grids (Plano GmbH), and stained with UranyLess and lead citrate (Electron Microscopy Sciences). Specimens were viewed on a FEI-Morgagni TEM operating at 80 kV.

1. Buchfink B, Reuter K, Drost H-G. 2021. Sensitive protein alignments at tree-of-life scale using DIAMOND. Nat Methods 18:366–368.
2. Chen S, Zhou Y, Chen Y, Gu J. 2018. fastp: an ultra-fast all-in-one FASTQ preprocessor. Bioinformatics 34:i884.
3. Dobin A, Davis CA, Schlesinger F, Drenkow J, Zaleski C, Jha S, Batut P, Chaisson M, Gingeras TR. 2013. STAR: ultrafast universal RNA-seq aligner. Bioinformatics (Oxford, England) 29:15–21.
4. Prjibelski A, Antipov D, Meleshko D, Lapidus A, Korobeynikov A. 2020. Using SPAdes De Novo Assembler. Curr Protoc Bioinformatics 70:e102.
5. Langmead B, Wilks C, Antonescu V, Charles R. 2019. Scaling read aligners to hundreds of threads on general-purpose processors. Bioinformatics 35:421–432.
6. Samson S, Lord É, Makarenkov V. 2022. SimPlot++: a Python application for representing sequence similarity and detecting recombination. Bioinformatics 38:3118–3120.
7. Zuker M. 2003. Mfold web server for nucleic acid folding and hybridization prediction. Nucleic Acids Res 31:3406–3415.
8. Vázquez AL, Alonso JMM, Parra F. 2000. Mutation Analysis of the GDD Sequence Motif of a Calicivirus RNA-Dependent RNA Polymerase. Journal of Virology 74:3888.

Table S1: Results of the bioinformatics virus discovery pipeline for PoBastV CHE/2022 and PoBastV AUS/2015

Scaffold	Length	Kmer-coverage	Best hit (GenBank accession number)	Identity	Alignment length
Porcine bastrovirus CHE/2022					
1	610 nt	x11	BLASTx:<br>Porcine endogenous retrovirus, envelope protein (CAB86207.1)	99.4%	170 aa
			BLASTn:<br>Porcine endogenous retrovirus Type D (HQ540593.1)	99.8%	555 nt
2	6068 nt	x327	BLASTx:<br>Porcine bastrovirus. nonstructural polyprotein (QDA77022.1)	93.3%	1206 aa
			BLASTn:<br>Porcine bastrovirus JPN/Iba-Ohata2/2015 (LC577872.1)	83.7%	5905 nt
Porcine bastrovirus AUS/2015					
1	1985 nt	X5	BLASTx:<br>Porcine bastrovirus. nonstructural polyprotein (UM077999)	99%	651 aa
			BLASTn:<br>Porcine bastrovirus JPN/Iba-Ohata2/2015 (LC577872.1)	85%	1981 nt
2	696 nt	X5	BLASTx:<br>Porcine bastrovirus. nonstructural polyprotein (BCL01237)	98%	232 aa
			BLASTn:<br>Porcine bastrovirus isolate BSF3(OM104033)	85%	693 nt
3	582 nt	X8	BLASTx:<br>Porcine bastrovirus. nonstructural polyprotein (BCL01237)	98%	193 aa
			BLASTn:<br>Porcine bastrovirus isolate BSF3(OM104033)	84%	582 nt
4	838 nt	X13	BLASTx:<br>Porcine bastrovirus. nonstructural polyprotein (UM077999)	96%	203 aa
			BLASTn:<br>Porcine bastrovirus isolate BSF3(OM104033)	89%	838 nt
5	1929 nt	X16	BLASTx:<br>Porcine bastrovirus. structural polyprotein (UM078000)	98%	503 aa
			BLASTn:<br>Porcine bastrovirus isolate BSF3(OM104033)	88%	1825 nt

aa: amino acids, nt: nucleotides

Table S2: Size of ORFs of different PoBastVs [number of nucleotides]. GenBank accession numbers are resolved in Figure S3.

	ORF 1	ORF 2	ORF 3A	ORF 3B	ORF 4
LC549662	3798	2046	tr.	tr.	588
LC577870	tr.	tr.	tr.	tr.	588
LC577871	tr.	2064	tr.	tr.	594
LC577872	3798	2046	336	264	588
MK387176	3798	2052	tr.	tr.	594
NC032423	tr.	2046	tr.	tr.	588
OM104033	tr.	2040	tr.	tr.	594
PP999616	3798	1941	336	264	588
PP999617	3798	1962	336	264	594

tr.: truncated

Table S3: Pairwise sequence identities [%] of ORF 3A/ORF 3B (grey fields) and ORF4 (white fields) sequences of PoBastVs. GenBank accession numbers are resolved in Figure S3.

	LC549662	LC577871	LC577872	MK387176	NC032423	OM104033	PP999616	PP999617
LC549662	-	88	92	88	92	88	90	86
LC577871	85/88	-	90	91	89	91	88	91
LC577872	97/97	84/88	-	88	94	89	90	87
MK387176	91/92	98/97	91/93	-	88	91	88	90
NC032423	96/96	84/86	97/97	92/94	-	88	89	87
OM104033	96/97	85/88	98/98	92/94	98/ 98	-	97	98
PP999616	96/97	84/89	96/97	92/93	95/95	96/97	-	86
PP999617	97/97	85/89	97/98	92/94	96/97	97/98	96/97	-

Table S4: PoBastVAUS/2015 RT-qPCR results in different organs of pig AUS/1

Sample Type	RT-qPCR result (cq)
Whole blood	22.5
Spinal cord*	17.7
Cerebrum*	22.3
Brainstem*	17.5
Brain stem and cerebrum*	17.8
Liver, kidney, heart, spleen*	31.4
Skeletal muscle, lymph node, lung*	31.6
Intestine, stomach, & pancreas*	29.8

*formalin-fixed paraffin-embedded tissue

Table S5: Primers used for RT-qPCR of PoBastV CHE/2022 and PoBastV AUS/2015

Virus	Primer sequence
CHE/2022 RT-qPCR	5’-AGC TGA TCA TTG GCG GAA CA-3’
	5’-FAM-TGC GCT ACA CTC GTT CTC AG-BHQ1-3’
	5’-GCG TTT GAA AGC TGC ACT GA-3’
AUS/2015 RT-qPCR	5′-GGTTCGCCTCACTCTGTTCAG -3
	5′-FAM-AGCAGCAAGCCATACGCCGTGC-BHQ1-3
	5′-CTCCCTGGGTACAGTCTTGCA -3′
CHE/2022 5’RACE	5′- AATTCTGCATTGCACGGAT -3′ (RT and first PCR)
	5′- CAATATCTATCTGCCACGCA-3′ (nested PCR)
CHE/2022 3’RACE	5′-TTTACATGCTCGCCTCAC-3′ (first PCR)
	5′-TGGCAGTTACAACTTCATGA-3′ (nested PCR)
CH/2022 ΔGDD mutagenesis	5′-CCATCTGATGTACGTGGATT-3′
	5′-CGAATGATTTGAACGCTGCACAGGCC-3′
